# Implications of microbe-derived ɣ-aminobutyric acid (GABA) in gut and brain barrier integrity and GABAergic signaling in Alzheimer’s disease

**DOI:** 10.1080/19490976.2024.2371950

**Published:** 2024-07-15

**Authors:** Kathryn A. Conn, Emily M. Borsom, Emily K. Cope

**Affiliations:** a Center for Applied Microbiome Sciences, The Pathogen and Microbiome Institute, Northern Arizona University, Flagstaff, AZ, USA; b Department of Biological Sciences, Northern Arizona University, Flagstaff, AZ, USA; c Center for Data-Driven Discovery for Biology, Allen Institute, Seattle, WA, USA

**Keywords:** Alzheimer’s disease, gut microbiota, gut-microbiome-brain axis, GABA, *Bacteroides*, *Lactobacillus*, tight junctions, blood brain barrier

## Abstract

The gut microbial ecosystem communicates bidirectionally with the brain in what is known as the gut-microbiome-brain axis. Bidirectional signaling occurs through several pathways including signaling via the vagus nerve, circulation of microbial metabolites, and immune activation. Alterations in the gut microbiota are implicated in Alzheimer’s disease (AD), a progressive neurodegenerative disease. Perturbations in gut microbial communities may affect pathways within the gut-microbiome-brain axis through altered production of microbial metabolites including ɣ-aminobutyric acid (GABA), the primary inhibitory mammalian neurotransmitter. GABA has been shown to act on gut integrity through modulation of gut mucins and tight junction proteins and may be involved in vagus nerve signal inhibition. The GABAergic signaling pathway has been shown to be dysregulated in AD, and may be responsive to interventions. Gut microbial production of GABA is of recent interest in neurological disorders, including AD. *Bacteroides* and Lactic Acid Bacteria (LAB), including *Lactobacillus*, are predominant producers of GABA. This review highlights how temporal alterations in gut microbial communities associated with AD may affect the GABAergic signaling pathway, intestinal barrier integrity, and AD-associated inflammation.

## Introduction

The gut microbiota, which is the aggregate of all microbial life in the gastrointestinal tract (GI), contributes to host physiologic functions through production of metabolites,^1,2^ neurotransmitters,^1,3–5^ and digestion of food.^6,7^ Communication between the brain and gut microbiota is known as the gut-microbiome-brain axis and functions bidirectionally via pathways such as the vagus nerve^8–10^ immune activation^11–13^ and circulation of microbial metabolites.^9,14^ Alterations of the gut microbiota and associated metabolites are implicated in a myriad of diseases including those related directly to gut health, such as celiac^12^ and Crohn’s disease,^15,16^ and neurodegenerative diseases such as Parkinson’s disease^17,18^ and Alzheimer’s disease (AD).^19–22^

AD is a debilitating neurodegenerative disease and the most common cause of dementia, affecting approximately 1:9 Americans over the age of 65^23^. Hallmark pathologies of AD include amyloid-β plaques, neurofibrillary tangles, and neuroinflammation.^23^ Factors contributing to AD pathogenesis are complex. Age is the greatest risk factor for AD and others include genetics, lifestyle, education, social engagement, obesity, diabetes, and traumatic brain injury.^24^ Recently, scientists have begun to understand that the gut microbiome, which is affected by genetics and lifestyle factors, is also a risk factor for AD. Several recent studies have demonstrated altered gut microbial composition in humans and in animal models of AD pathologies.^19–22,25^

Studies of the gut microbiota in AD in both human and animal models indicate a shift in the gut microbiota composition, preceding and concomitantly with onset of pathologies.^19,20,22,26^ Several studies find *Bacteroides* differentially abundant in the fecal microbiota of both humans and animal models of AD pathologies.^19,21,27,28^ Borsom et al. demonstrated that *Bacteroides acidifaciens* was enriched after pathological onset in triple transgenic mice modeling AD pathologies (3×Tg-AD) whereas *Lactobacillus salivarius* in 3×Tg-AD mice was depleted compared to WT controls, prior to emergence of characteristic pathologies.^22^ Changes in the gut microbial communities may be a direct result of changing neurochemical balances within the host,^29^ leading to greater demand on microbes themselves to create and regulate neurotransmitters as energy sources. Key gut microbial taxa, including those altered in AD, such as *Bacteroides* and *Lactobacillus*, are known to produce the mammalian inhibitory neurotransmitter, ɣ-aminobutyric acid (GABA).^4,9,30^ The role of GABA in the gut in AD may include stimulating the vagus nerve to send afferent signals to the brain or altering gut mucosal membrane barrier integrity through changes in mucin production.^9,31,32^ The mucus layer in the GI tract forms a physiologic barrier between microbes and gut epithelia and facilitates the movement of solutes within and out of the gut.^9,33^ Reduced mucus layers allow closer proximity of microbes and their metabolites to the gut epithelial cells, compromising tight junctions and allowing passage of microbial products into the lamina propria.^34–36^ To this end, endogenous GABA produced into the gut may escape through the intestinal epithelia,^37^ into the lamina propria, and travel through the bloodstream to the brain.

Tight junctions are a complex network of proteins which regulate gut epithelial and blood–brain barrier (BBB) integrity and modulate the movement of solutes across cell barriers.^38–40^ Some studies suggest that gut barrier integrity may be compromised in AD,^36,41^ which may allow microbial metabolites and byproducts into the lamina propria and access to the bloodstream.^42–44^ Regulation of tight junction proteins occurs through host and microbial metabolites and host inflammatory processes.^38,40,45^ Dysregulated tight junction proteins may allow the translocation of host- and microbe-derived metabolites to escape into the bloodstream and reach the BBB, which is also damaged in AD.^46–49^ Increased intestinal permeability, coupled with neuroinflammation affecting the integrity of the BBB, may be an important pathway in the gut-microbiome-brain axis. The goal of this review is to highlight how alterations in the gut microbiota in AD influence intestinal barrier integrity, with a focus on the emerging role of microbiome-derived neurotransmitters in signaling through the GABAergic pathway.

## Alzheimer’s disease

AD is a neurodegenerative disease characterized by amyloid-β plaques and neurofibrillary tangles and is the most common cause of dementia among elderly individuals (~60–80% of dementia cases) in the United States.^50^ While the incidence of AD appears to be declining,^51^ the population of people living with AD continues to rise with the planet’s growing number of aging individuals.^50^ Symptoms of this disease include memory loss, trouble with fine motor movement, behavioral changes, and eventually, death.^50,52^ While each of these symptoms are common manifestations of aging, even in healthy individuals, patients living with AD experience these symptoms at greater severities than what would be expected for their age.^52^ Behavioral changes often accompanying AD include depression, anxiety, fear, and paranoia.^52^ These symptoms may be observed by family, friends, and caregivers prior to a formal diagnosis; AD pathogenesis is thought to begin decades before the development of behavioral changes.^52^ Accordingly, recent research suggests that pathological changes in brain and cerebral spinal fluid chemistry may occur up to 25 years prior to cognitive decline.^53,54^

Key characteristics of AD are aggregation of amyloid-β plaques, neurofibrillary tangles, neuroinflammation, and BBB breakdown.^46^ Amyloid-β is naturally produced in the brain, first as amyloid precursor protein (APP).^55^ In a healthy brain, presenilin proteins (PSEN), presenilin-1, and presenilin-2, cleave APP most often into amyloid-β40 (~90%).^55,56^ Commonly in familial AD, mutations in APP or in PSEN cause cleavage of APP into amyloid-β42. The amyloid-β42 peptide is more hydrophobic and the most common isoform of amyloid-β plaques.^55–58^ These insoluble peptides aggregate in the brain, forming amyloid-β plaques, and are associated with neurotoxicity.^55,57,59^ Neurofibrillary tangles caused by breakdown of the stabilizing protein Tau follow amyloid-β plaque onset.^29,60^ In AD, Tau is hyperphosphorylated, leading these proteins to dissociate from the neuron and tangle on other tau proteins.^29,54,60^ This causes neurons to collapse and prevents communication between synapses. Despite being well characterized in AD, therapeutics targeting amyloid or tau pathologies have had limited success in human clinical trials^61–63^ so understanding how peripheral factors, such as the gut microbiota, contribute to pathology development is critical.

Breakdown in the BBB is implicated in AD and is thought to begin in the hippocampus, which modulates long-term memory, learning, and emotional processing, preceding cognitive decline.^49^ Compromised integrity of the BBB can allow translocation of toxic materials such as circulating iron into the brain.^49,64^ BBB integrity is maintained by a network of tight junction proteins including occludin and several claudins.^65,66^ Damage to the BBB may precede hallmark amyloid-β plaques and tau tangles,^47^ or may be a consequence of pathological onset.^46,49^ As few studies have investigated the effect of AD pathologies on tight junction protein and mRNA expression, future research should aim to identify which and how proteins are being compromised due to AD. The AD-associated gut microbiota can drive disease pathologies by increasing inflammation, signaling through the vagus or enteric nervous system, or by compromising gut barrier integrity and allowing microbial products to reach the compromised BBB.^28,32,36,67,68^

## Dysregulation of GABAergic signaling in AD

GABA is a downstream product of the excitatory amino acid, glutamate. Glutamate can be produced in the brain via astrocytes and is also a component of human diets.^69–71^ The gut and associated microbes have a high capacity for dietary glutamate; it is converted into several important molecules in the gut including proline, arginine, glutamine, and GABA.^71,72^ The enzyme, glutamate decarboxylase (GAD), catalyzes the transformation of glutamate into GABA.^30,73^ The GAD gene is found in the genomes of several key AD-associated bacteria including *Bacteroides, Bifidobacterium, Alistipes, Blautia*, and *Lactobacillus*.^74^ In AD patients, low plasma levels of glutamate are associated with lowered ability to name objects, follow directions, and behavioral symptoms.^75^ Furthermore, human patients and animal models of AD exhibit decreased levels of glutamate in the brain.^76,77^ By contrast, glutamine, the precursor of glutamate, is found elevated in the plasma of AD patients.^78^ The glutamate concentration in the gut, as it pertains to AD, has not yet been investigated. Future research in AD should seek to close the knowledge gap existing about glutamate/GABA levels in the gut of AD patients and mouse models.

Correlated with AD development are obesity and diabetes; diseases which can also be affected by microbe-derived GABA or bacterial GAD genes. Supplementation with GABA in mice fed high fat diets promotes fat browning, which is involved in thermoregulation as opposed to energy storage, and regulates lipid metabolism.^79,80^ Transplantation of fecal material from lean donors into metabolic syndrome recipients has been shown to improve insulin sensitivity, altered microbiome composition, and changes in plasma metabolites including GABA.^81^ Thus, GABA’s role in AD may be peripheral or may affect both AD and associated comorbidities. In type-1 diabetes, *in silico* analysis suggests that the gut microbiome may become depleted of bacteria expressing the GAD gene.^82^ As GAD-expressing bacteria die, they can release bacterial GAD, which mimics human GAD, and is recognized as an antigen in the gut by type-1 diabetes autoantibodies.^82^ Bacterial GAD then activates submucosal T cells which subsequently destroy pancreatic beta cells expressing the human GAD enzyme.^82^ As type-1 diabetes is positively correlated with the development of AD,^83,84^ it is plausible that the dysregulation of the GABAergic system in AD is not due to GABA itself but the genes involved in its production.

Recent studies indicate that GABAergic signaling is dysregulated in AD.^85–87^ In one study, GABA concentration in AD and age-matched cognitively normal participants demonstrated decreased GABA/creatine ratios in the parietal, but not frontal, region in patients experiencing AD as compared to cognitively normal controls.^88^ No significant differences were observed, however, between GABA concentration and the results of the Mini Mental Status Examination, suggesting the GABAergic pathway may not be implicated in cognitive manifestations of the disease.^88^ These results contrast a study on cerebrospinal fluid levels of GABA which found that GABA was more abundant in study participants experiencing mild AD as compared to three other groups ranging from subjective cognitive impairment, used as control, to participants experiencing cognitive impairment which would progress to AD within 2 y.^86^ These differences may, in part, be attributable to a difference in GABA transport mechanisms as opposed to levels of GABA itself. In postmortem brain tissue analysis of probable and definite AD diagnoses, researchers found region-specific inhibition in GABA reuptake transporter protein expression in the hippocampus, subiculum, entorhinal cortex, and superior temporal gyrus in AD.^89^ Inhibition of GABAergic signaling has also been studied in several strains of mice modeling AD pathologies.^90–92^ In hippocampus samples collected from a mouse model of AD, researchers determined that reactive astrocytes, which gather around amyloid-β plaques, produce high levels of GABA, resulting in impaired synaptic plasticity, learning, and memory.^93^ GABA is further implicated in AD as administration of GABA_A_ receptor antagonists, bicuculline, and/or picrotoxin can remediate cognitive deficits in mouse models of AD pathologies.^94^ Interestingly, this study demonstrated age-related alterations in the GABAergic pathway regardless of strain, which indicates that changes are an intrinsic part of aging.^94^ Little is known about the implications of gut-derived GABA on AD disease pathogenesis and its role in the bidirectional communication between the brain and gut in AD.

## The gut microbiota is altered AD

The gut microbiome assists the body in digestion, production of neurotransmitters, and microbial metabolites and is a contributor to healthy aging or age-related decline.^95^ The gut microbiome undergoes dynamic temporal changes throughout the lifespan in response to host and environmental factors.^96^ An analysis of the gut microbiome of 9,000 individuals between the ages of 18 and 101 demonstrated some interesting patterns associated with health and longevity. One pattern that emerged in healthy aging was depletion of *Bacteroides* later in life. High abundance of *Bacteroides* in older adults (over 80) was predictive of decreased survival.^96^ Aging is also associated with increased uniqueness of the gut microbiome, or high interpersonal variability.^95,96^ However, it is difficult to generalize patterns across studies because of confounding effects such as variability in study designs, altered diets between elderly and young adults, and living environments. Generally, as humans age into elderly adults, the gut microbiome decreases in diversity and demonstrates taxonomic changes with microbial taxa that are often associated with altered metabolic profiles and disease phenotypes.^97–99^ In AD, recent literature has consistently demonstrated that altered microbial communities are correlated with disease state (preclinical, mild cognitive impairment, or cognitively impaired AD). Generally, the gut microbiota in patients living with AD is less diverse and taxonomically distinct when compared to age- and sex-matched controls.^19,20,100,101^ In this review, we focus on the role of *Bacteroides* and Lactic Acid Bacteria (LAB)^102–104^ because these taxa have previously been identified as differentially abundant in human and mouse studies of AD and AD pathologies,^19,21,22,28^ and are the predominant producers of GABA in the human gut microbiome.

Gut microbiome alterations may precede cognitive decline in AD. A recent study identified bacterial taxa associated with preclinical AD, when patients are cognitively normal but biomarkers of AD are present.^105^ In this study, members of the genus *Dorea, Oscillibacter*, and *Faecalibacterium* were enriched in patients with preclinical AD, while members of the genus *Bacteroides* were more prevalent in healthy participants.^105^ In another study of individuals with mild AD compared to age- and sex-matched cognitively normal participants, *Bacteroides* were enriched in AD, and LAB (in this study, *Lactococcus*) were enriched in healthy participants, independent of dietary factors.^19^ Mouse studies demonstrate alterations in similar taxa. In a longitudinal study of 3×Tg-AD mice modeling AD pathologies, we found enrichment of *Bacteroides* species after the onset of amyloid-β plaque formation, and *Lactobacillus* species were enriched in wild-type control mice at early timepoints,^22,28^ but this trend is not consistent and is likely dependent on species and strain. A longitudinal study using metagenomic sequencing found that several species of *Lactobacillus* were enriched in 5×FAD mice, but *L. johnsonii* were less abundant.^28^ These findings indicate a potential role for GABA-producing microbiota in AD.

Current strategies targeting the gut microbiota to yield mechanistic insights into AD typically colonize the gut microbiota with species or microbial communities of interest via fecal microbiota transplantation (FMT). Several FMT studies in animal models of AD indicate that the gut microbiome can reverse cognitive decline and reduce disease pathologies, either through direct methods to transfer fecal microbiota or cohousing mice modeling AD with WT controls.^106–108^ When WT mice are cohoused with 5×FAD mice for the first seven months of life, the gut microbiota was transferred via coprophagy and resembled an intermediate phenotype with some features of a WT and some features of a 5×FAD microbiome.^109^ This led to a brain transcriptome resembling 5×FAD mice, increased infiltrating Th1 cells into the brain, and activated microglia in co-housed WT mice compared to mice WT caged with their genotype.^109^ Likewise, a study in which FMT was directly administered from transgenic mice modeling AD pathologies into wild-type mice demonstrated microglia activation and colonic inflammation when compared to the wild-type mice receiving vehicle control or FMT from other wild-type mice.^110^ In humans, FMT treatment has shown promising results in other neurological disorders, most prominently in studies of children with autism^2^ and one case study of an AD patient demonstrated rapid improvement in cognitive function following FMT for *Clostrium difficile* infection.^111^ Currently, there is no cure or preventative therapeutics for AD, but promising research into the gut microbiome offers the potential for customized therapeutics targeting distinct niches of the gut microbiome.

### Bacteroides in AD

*Bacteroides* are Gram negative, anaerobic bacteria commonly found colonizing the colon. Gram negative bacterial cells express lipopolysaccharide (LPS) on the cell surface, a compound which is known to induce inflammation and can act on tight-junction proteins in the gut through regulation of inflammatory cytokines.^2,7,112^ In a study of germ-free 3×Tg-AD mice humanized with fecal microbiota from AD and healthy participants, *Bacteroides* was enriched in mice receiving AD-associated fecal microbiota, and correlated with increased microglial activation.^113^ Another study, using Thy1-C/EBPβ transgenic mice that overexpress EBPβ, a critical transcription factor for APOE, humanized mice with fecal samples from AD and age-matched donors. They demonstrated increased abundance of *Bacteroides fragilis* when treated with AD fecal microbiota. Levels of *B. fragilis* in the gut correlated with elevated serum and brain levels of 12-hydroxy-heptadecatrienoic acid (12-HHTrE) and Prostaglandin E2, metabolites known to stimulate microglia activation. Furthermore, mice treated with AD donor stool demonstrated an increase in brain levels of polyunsaturated fatty acid enzymes, cyclooxygenases, COX-1, and COX-2, which promote prostaglandin E2 synthesis.^114^ In the brain, COX-1 is expressed predominantly by microglia whereas COX-2 is expressed by hippocampal neurons.^114^ As *Bacteroides* are often, but not always, enriched in humans and animal models of AD pathologies, understanding its effects on the brain in AD, especially microglia activation, can help us better understand the bidirectional pathways that lead to its enrichment or depletion in the gut microbiome.

The molecular mechanism underlying *Bacteroides* effects in AD are not yet known but are likely multifactorial, and may include inflammation due to the release of LPS, alteration of microbiome composition through metabolic cross feeding, and production of GABA. *Bacteroides fragilis* LPSs are structurally unique and are potent activators of proinflammatory pathways in primary human brain cells *in vitro*.^115,116^ LPS administered to CD-1 mice, which are outbred multipurpose mice, via intraperitoneal injection exhibit altered amyloid-β transport at the BBB. This resulted in increased burden of amyloid-β in the brain and increased neuronal expression of LRP-1, a protein which mediates amyloid-β uptake into neurons, astrocytes, and microglia.^48^ The effects of LPS were likely a consequence of increased inflammation rather than a direct effect on BBB cells; serum levels of IL-6, IL-10, IL-13, and major cationic protein (MCP)-1 were found upregulated and corresponded to amyloid-β influx into the brain.^48^ Increased LPS production by gut microbes may stimulate local inflammation, altering gut epithelial tight junctions similar to BBB tight junctions, and escape into the bloodstream or interstitium and reach the brain.^68,117^ Finally, *Bacteroides* are known producers of the inhibitory neurotransmitter, GABA (discussed in detail below).^30^ Very little research has been done on microbe-derived neurotransmitter effects on neurodegeneration although there are strong indications of alterations in the GABAergic pathway in AD.^88–120^ The effects of *Bacteroides* in the gut may also be indirect, as introduction of one bacteria may modulate the microbial community structure as a whole,^121,122^ wherein *Bacteroides* acts as a keystone species in the gut microbial ecosystem.

### Lactobacillus in AD

*Lactobacillus* is a Gram positive, facultative anaerobe, and is commonly studied for its potential for neuroprotection. *Lactobacillus* is a primary producer of lactic acid. Two enantiomers of lactic acid exist. L-lactic acid is a common host metabolite, but D- and L-lactic acid can be produced by strains of *Lactobacillus* in the host microbiome.^123,124^ L-lactic acid is associated with health, but D-lactic acid has been demonstrated to have some toxicity to the host. In the brain, accumulation of D-lactic acid is associated with memory impairment and neuronal inhibition due to competitive blocking of L-lactic acid uptake.^125^ In addition, similar to *Bacteroides, Lactobacillus* are emerging as key GABA producers^4,9,104^ Given that species of *Lactobacillus* produce both GABA and L-/D-lactic acid, the findings discussed below may be driven by either or both metabolites. Since this review is focused on GABA, we narrowed our discussion to neurotransmitter activity.

Several studies indicate protective effects of taxa within the genus *Lactobacillus* against neurodegeneration or cognitive deficits.^102,122,126,127^ The effects of *Lactobacillus* may be direct or indirect. Supplementation of exogenous *Lactobacillus* into the gut microbiome can alter the overall microbiome composition, modulate intestinal permeability, and gut epithelial tight junction integrity.^102,128^ Intervention with exogenous *Lactobacillus plantarum* PS128 is protective in a 3×Tg-AD mouse model.^102^ Mice in this study were treated either with *L. plantarum* PS128 or vehicle control for one month before receiving an intracerebroventricular injection of either streptozotocin (which exacerbates AD pathologies in this model) or vehicle control. Mice receiving *L. plantarum* PS128 were protected against streptozotocin-mediated damage, characterized by reduced amyloid deposition, tau hyperphosphorylation, gliosis, and astrocytosis compared to the untreated group.^102^ The authors suggest that gliosis in this model is mediated by fecal propionic acid and GSK3β levels, and that *L. plantarum* P128 regulates fecal propionic acid.^102^ In a mouse model of anxiety and depression, administration of *Lactobacillus* reduced anxiety and depression symptoms which correlated with altered GABA receptor expression in several brain regions including the hippocampus.^9^ These effects were lost when the vagus nerve, which innervates the gastrointestinal tract, was cut, suggesting afferent vagal signaling as a mode of bidirectional communication between gut microbes and the brain.^9^ While more research, particularly in human participants, is necessary, exogenous *Lactobacillus* may confer a health benefit in neurological disease.

Endogenous *Lactobacilli* can also have a role in health status. Borsom et al. demonstrated depletion of *Lactobacillus salivarius* in mice modeling AD pathologies prior to pathology development, possibly indicating that gut microbiota at a preclinical stage may play an important role in neuroprotection.^22^ Another study demonstrated depletion of *L. johnsonii*, but enrichment of *Lactobacillus* P38 in 5×FAD mice compared to controls.^28^ In AD, it is conceivable that increased production of GABA and/or lactic acid L- and D-enantiomers by *Lactobacillus* during preclinical stages of disease inhibits vagus nerve stimulation or otherwise affects gut epithelial tight junctions and that this may have downstream effects on disease pathogenesis.

## Tight junction protein regulation by the microbiome

The integrity of the intestinal epithelial layer is carefully maintained through host- and microbe-derived metabolites and cytokines. In a healthy gut ecosystem, the gut epithelial cells do not come into direct contact with the microbes due to a highly glycosylated mucus layer secreted by goblet cells. A compromised epithelial barrier can allow translocation of metabolites that otherwise would not cross this highly regulated barrier.

Several gut taxa within the genera *Akkermansia, Bacteroides, Prevotella, Clostridium*, and *Streptomyces* are known degraders of mucins, meaning they can use mucin as a carbon source and this thins the gut mucus layer.^129–131^ In a healthy gut ecosystem, gut bacteria mediate a dynamic equilibrium of the gut mucus layer which prevents direct contact with gut epithelial cells but allows movement of microbe-derived products to the host.^132^ While each of the aforementioned taxa can use mucins as their carbon/nitrogen sources, complete degradation of mucin relies on a diverse community of microbiota and associated mucin-degrading enzymes, collectively known as “mucinases”.^129^ In a disease-associated gut microbiome, enrichment of mucin-degrading bacteria could plausibly degrade the mucus layer faster than it is replenished and damage the gut epithelial cells or associated tight junction proteins.

The tight junction is composed of several transmembrane proteins, including claudins, occludins, and zonula occludens, which interact to maintain epithelial barrier integrity.^133^ Mice with knockout tight junction proteins such as claudins and occludins show severe immune infiltration and chronic inflammation along with hyperplasia of the gastric epithelium in the occludin knockout mice,^134^ and claudin-7 knockout mice died in the perinatal period.^135^ These results underscore the critical role that these proteins play in gut barrier functioning and the consequences of their dysregulation. Tight junction protein dysregulation is implicated in myriad gut and neurological disorders including metabolic disorders, inflammatory bowel disease, Crohn’s disease, and autism spectrum disorder (ASD).^136–138^ The role of intestinal barrier function in AD is less well understood. Loss of intestinal barrier integrity has been observed in 5×FAD and APP/PS1 models of AD pathologies,^36,45^ and in some small human studies of preclinical AD and dementia.^139^ Gut microbiota play a critical role in maintaining barrier integrity. In a mouse model of ASD, researchers challenged mice with *B. fragilis* every other day for 6 d immediately after weaning.^2^ Results from this study indicate that *B. fragilis* corrects intestinal barrier integrity and restores tight junction proteins. *B. fragilis* also corrected behavioral deficits in mice modeling features of ASD.^2^ Taken together, these data suggest that *B. fragilis* may modulate the gut-microbiome-brain axis through regulation of tight-junctions proteins and related cytokines and/or through the regulation of other enteric microbiota which then influence expression of tight junctions and inflammatory cytokines.

The BBB, which maintains the delicate influx of necessary nutrients into the brain while blocking neurotoxic proteins, metals, immune cells, and microbes, is also compromised in AD.^40,46,49,66^ The BBB, similar to the gut, is maintained by tight junction proteins, claudins and occludins.^39,65^ BBB degeneration is found even in healthy brains as people age, but degeneration is accelerated in AD and other progressive, neurodegenerative diseases.^49^ This may be due to infiltration of blood-born macrophages which appear to be recruited by neuronal cells experiencing amyloid-β aggregation.^40^ Here, we explore whether compromised intestinal and BBB integrity may constitute a mechanism by which the gut microbiome affects AD.

Emerging evidence suggests that the gut microbiome and intestinal barrier integrity are altered in AD.^36,45,139^ Studies have demonstrated decreased gut tight junction protein integrity measured by decreased levels of occludin, ZO-1, and claudin1 mRNA and protein expression in humans and mouse models of AD.^36,140^ These observations led us to explore this as a potential mechanism contributing to disease progression. A compromised tight-junction layer in the gut epithelium could allow translocation of gut- and host-derived metabolites which, in turn, act on the brain. A compromised BBB integrity may then expose the brain and associated inflammatory cells to an exogenous, gut derived metabolic milieu. Thus, damage to tight junctions in the gut epithelial layer may be an unseen pathology of neurodegenerative diseases.

## Effect of *Bacteroides* on barrier integrity

Several studies investigating the role of the gut microbiota in neurological diseases point to the genus, *Bacteroides*, as a potential neuroactive member of the gut microbiome.^2,141^ *Bacteroides* can act through several mechanisms, including production and translocation of LPS from the lumen to the bloodstream, in the context of a damaged epithelial barrier.^8,142^ LPS in the bloodstream, a condition known as metabolic endotoxemia,^143^ is known to stimulate inflammation of both the gut and peripheral organs, including the brain.^7,8,112,117^ The role of Gram negative, LPS-producing bacteria remains controversial as other research, examining the role of *Bacteroides fragilis* in mice modeling Autism Spectrum Disorder (ASD), indicates *Bacteroides* remediates damage to gut epithelial tight-junctions through regulation of claudins (CLDNs 8 and 15).^2^ *Bacteroides* can use gut mucins as a carbon source.^144^ In the context of a healthy microbiome, this can help maintain a healthy dynamic equilibrium between mucin production and degradation, but in the context of disease could lead to direct interaction with the epithelial layer and compromise barrier integrity.^35,130^ The specific effect of *Bacteroides* in AD is poorly understood and is likely strain-specific. Implications of *Bacteroides* regulation of intestinal barrier integrity in AD may be beneficial, increasing expression of tight junction proteins, or detrimental through degradation of the mucosal layer. Additional research is needed to characterize the strain-specific effects of *Bacteroides* on gut barrier integrity in AD.

## Effect of *Lactobacillus* on barrier integrity

*Lactobacillus* are known to confer benefits to human hosts through resistance to pathogenic invasion,^145^ gastrointestinal motility and mucin production,^146^ tight junction regulation,^128,147,148^ and neurotransmitter activity.^4,102,104,146^ Several studies indicate that administration of *Lactobacillus* improves gut barrier integrity through an increase in gene and protein expression of occludin, a tight junction protein important in gut barrier stability and function.^128,147,149,150^ Administration of *Lactobacillus plantarum* MB452 to cultured human colon cancer cells altered 19 genes involved in the tight junction regulation pathway.^149^ In particular, gene expression of occludin and occludin-associated plaque proteins, ZO-1, ZO-2, and cingulin were increased and this was associated with increased barrier integrity.^149^ Interestingly, of the 19 genes that were altered in the tight junction pathway, 2 of those genes, ITCH, and SNAI1, were involved in tight junction disassembly, suggesting that *L. plantarum* MB452 modulates the tight junction pathway as opposed to a single gene or protein.^149^

*Lactobacillus* is further implicated in gut health through modulation of gut mucin production, especially MUC-2,^146,151^ the dominant mucin in the gut. In mice modeling increased amyloid-β aggregation and cerebral amyloid pathology, mice at 6 months of age demonstrated decreased mucus maturation in comparison to an age-matched WT cohort but this same difference was not observed in mice with advanced pathologies (15 months)^41^, suggesting that shifts in gut mucus integrity may precede and potentially dictate future pathological onset. Furthermore, symptomatic mice in this study had an increased abundance of *Lactobacillus* in comparison to age-matched WT controls and this was not observed at the baseline stage.^41^ The potentially protective effects of *Lactobacillus* or other LAB are not well understood in AD. Further research needs to be done to evaluate their potential in modulating the gut epithelial barrier in preclinical stages of AD.

## GABA production by the gut microbiome; effects on mucosal barrier integrity, vagus, and enteric nerve stimulation, and neuroinflammation

**Figure 1.**
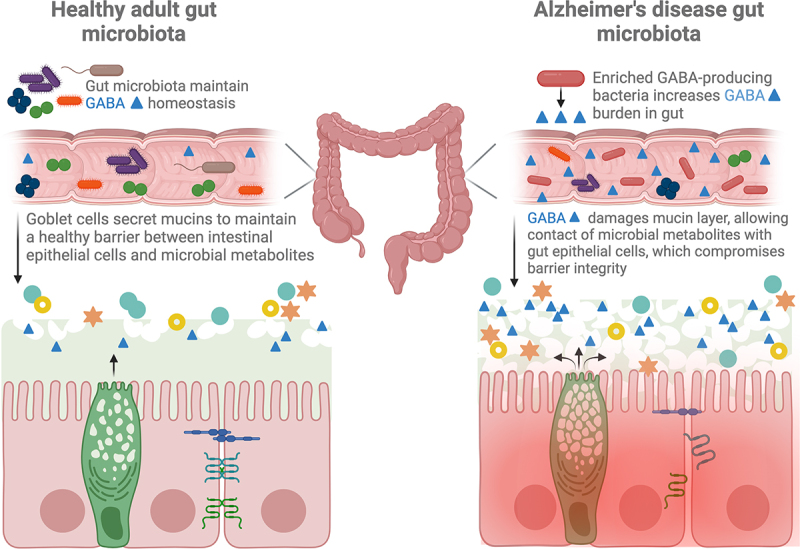
The gut mucus layer in AD is damaged by increased GABA-producing microbes in the context of a disease-associated microbiome. Damage to the mucus allows closer proximity of microbes and their metabolites to gut epithelial cells, damaging tight junction proteins and stimulating inflammation.

Neurotransmitters produced by the gut microbiota are important mediators of the gut-microbiome-brain axis, and this is emerging as a field of “microbial endocrinology”.^3,152^ Gut microbiome-derived neurotransmitter signaling pathways that are hypothesized to act along the gut-microbiome-brain axis include tryptophan metabolism, which affects both the serotonin and kynurenine pathways,^1,3,153^ and the GABAergic pathway,^3,9,14,30,154^ which inhibits excitation in the central nervous system. We focus here on microbial influence on GABAergic signaling; microbial modulation of other neurotransmitters and signaling pathways have been reviewed in.^155,156^ Several bacterial taxa are known producers of molecules implicated in the GABAergic pathway, including glutamate and GABA itself (Table 1).^3^ GABA and glutamate producing taxa encode Glutamate decarboxylase, which converts glutamate into GABA, and putrescine aminotransferase, which catalyzes the reaction that produces L-glutamate, the main excitatory neurotransmitter and precursor for GABA (see Table 1 for references). The local effects of GABA in the gut of people living with AD and animals modeling AD pathologies is poorly understood, although *in vitro* research in healthy gut cell lines indicates that GABA can significantly increase the expression of mucins.^31^ Other studies in live mice and cultured human colon cancer cells suggest that increased GABA may damage the mucus layer.^33,157^ Studies investigating the role of GABA on healthy animals or tissue indicate beneficial effects of GABA such as immune system regulation and increased intestinal integrity.^31,158^ Other studies in disease models such as colitis indicate that GABA may negatively affect host health through damage to the mucus layer.^33,157^ Damage to the mucus layer allows closer proximity of microbes and their metabolic byproducts to the gut epithelial layer, compromising tight junction proteins,^33,34^ and stimulating epithelial and systemic inflammation (Figure 1). Identifying the disease-specific role of GABA in AD will further our understanding of the bidirectional cross talk of the gut-microbiome-brain axis.

**Table 1.** GABA-producing microbiota with relevance to AD.

Bacterial genus	GABA production	Glutamate decarboxylase gene in bacterial chromosome	Putrescine aminotransferase gene in bacterial chromosome	Enriched in AD	Depleted in AD
*Bacteroides spp.*	+	+	–	19, 21, 22	26
*Lactobacillus spp.*	+	+	+	NA	22
*Bifidobacterium spp.*	+	+	+	26	19, 21
*Turicibacter spp.*	–	–	–	22, 26	19
*Akkermansia spp.*	+	+	–	21, 22, 26	NA
*Prevotella spp.*	potential	+	–	21, 22	NA
*Clostridium spp.*	–	–	+	NA	19, 21
*Adlercreutzia spp.*	–	–	–	NA	19, 21
*Alistipes spp.*	+	+	+	19	21, 26
*Blautia spp.*	+	–	–	19, 26	21

So far, few studies have investigated the interactions between GABA and tight junction protein expression as it pertains to AD. Gene and protein expression of claudin-5, one of the dominant tight junction proteins in the brain, is decreased in AD, and associated with cognitive decline.^32^ Cognitive decline is associated with decreased long-term potentiation of synaptic efficacy in the hippocampus, which is the means by which the brain is thought to strengthen synaptic connection and store memories.^159^ Knockout heterozygous claudin-5^±^ mice demonstrate impaired long-term potentiation due to enhanced GABAergic transmission.^32^ Administration of a GABA_A_ receptor antagonist in claudin-5^±^ mice remediates changes in long-term potentiation which further supports a causal role of claudin-5 mediated GABAergic transmission.^32^

GABA in the colon may act entirely differently than in the brain. In humans and mouse models of dextran sulfate sodium (DSS)-induced colitis, several studies indicate that GABA administration damages the mucus barrier of the colon, reduces tight junction protein expression, increases gut epithelial permeability, and induces colonic inflammation.^33,157^ Furthermore, GABA inhibited colonic epithelial cell proliferation when bound to GABA_A_ receptors,^33,157^ and previous research suggests that increased GABA receptor expression correlates with increased epithelial inflammation.^157^ While more research is needed to understand GABA’s effects on tight junction gene and protein expression in AD, these results underscore the interconnected nature of GABA and intestinal integrity.

**Figure 2.**
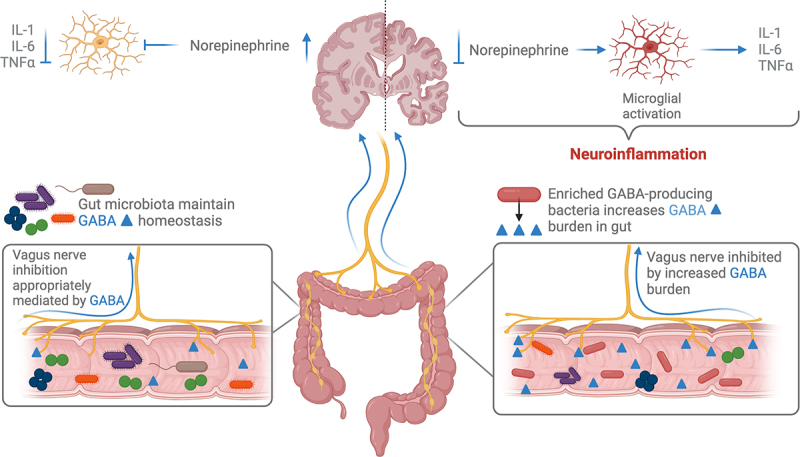
GABA-producing bacteria enriched in the disease-associated gut microbiome increase the local concentration of GABA. GABA inhibits the vagus nerve in the gut and afferent signaling to the brain downregulates the release of norepinephrine. Norepinephrine is responsible for inhibiting microglia activation and subsequent microgliosis.

Exogenously produced GABA by gut microbes may also act on the intestinal microenvironment and signal via afferent vagus nerve or enteric nerve pathways. Vagus nerve stimulation (VNS), which is currently used in the treatment of epilepsy, may be beneficial in AD. Vargas-Caballero et al. propose VNS for treatment of AD as its anti-inflammatory effects may modulate neuroinflammation.^160^ Evidence from animal and clinical studies show that VNS stimulation transiently increases GABA, serotonin, dopamine, and norepinephrine in the CSF and various brain regions including the hippocampus.^67,161,162^ In some studies, increased GABA or GABA receptor density following VNS is associated with improved neuroplasticity or behavior,^161,162^ but the number of studies assessing the exact mechanism are limited. Further, no studies have measured whether VNS changes the quantity of GABA in the gut. One study, however, demonstrated that vagotomy can increase GABA production in the gut.^163^ Zou and colleagues investigated the effect of vagotomy on susceptibility to intestinal infection and anxiety-like behaviors in a mouse model. In their study, vagotomy decreased the susceptibility to infection with *Salmonella*, reduced anxiety-like behaviors, and increased GABA production and GABA-producing microbes in the gut.^163^ Enrichment of GABA-producing bacteria in the gut of AD patients and mice modeling AD pathologies may inhibit VNS thus upregulating neuroinflammation (Figure 2). If gut microbes enriched in AD such as *Bacteroides* and *Lactobacillus* are effective at antagonizing VNS, gut microbial therapeutics may aim to reduce their abundance in the gut or recolonize the gut with more stimulatory microbes. The specific effects of the role of the vagus nerve in relation to microbe-derived GABA are discussed below, with a specific focus on *Bacteroides* and *Lactobacillus*.

The enteric nervous system (ENS) is composed of enteric neurons and enteric glial cells, and regulates GI motility, secretions, and the enteric immune system.^164,165^ Within the ENS, approximately 5–8% of the myenteric neurons, which regulate GI motility and mucosal function, contain GABA.^166,167^ In these enteric neuronal cells, GABA is likely involved in secretory and motor GI function.^165^ Enteric glial cells, which have similar function to the glial cells in the central nervous system, contribute to regulation of inflammation in the intestines. However, the role of intestinal GABA on regulating enteric glial cell function has not yet been extensively studied. A recent study found that enteric glial cells do express GABA signaling receptors, and that administration of GABA inhibits the pro-inflammatory NF-kB pathway in enteric glial cells in a model of intestinal inflammation.^168^ Microbe-derived GABA may well influence the function of the ENS and regulation of inflammation in the intestines, but future research is needed in this exciting but nascent field.

## *Bacteroides* production of GABA

*Bacteroides* are the predominant producers of GABA in the human gut. In a screen of 961 *Bacteroides* genomes, 95% of the human gut isolates of *Bacteroides* were found to contain the GAD genes, enabling *Bacteroides* to transform glutamate into GABA (Table 1).^30^ The same study evaluated *in vitro* production of GABA and found 16 distinct species of *Bacteroides* produced GABA at concentrations ranging from 0.09 to 60.84 mM.^30^ Functionally, GABA may contribute to acid stress tolerance for *Bacteroides* and has been implicated in metabolic cross-feeding in the gut microbiota with GABA consuming microbes.^30,169^ As discussed above, the composition of the gut microbiome is altered in AD, and *Bacteroides* is frequently identified as differentially abundant in disease. In the context of a disease-associated microbiome, it’s possible that GABA production by *Bacteroides* is also altered. If GABA is increased in a diseased state, the epithelial layer may be more susceptible to damage through GABA-mediated disruption of the mucus layer. Thus, GABA production by *Bacteroides* may be a double-edged sword in AD, as increased GABA signaling to the brain positively affects cognitive symptoms and molecular pathologies,^67^ but also disrupt the gut mucus layer and compromise the gut epithelial integrity.^33,157^

## *Lactobacillus* production of GABA

LAB are also predominant producers of GABA, including the species in the genus *Lactobacillus* .^170^ Many species in the genus *Lactobacillus* have been studied for their beneficial effects on host health in a variety of diseases and conditions.^4,104,145,150,171^ In the gut-microbiome-brain axis, the majority of research has focused on exogenous *Lactobacillus* mediated GABAergic signaling in Major Depressive Disorder (MDD).^4,9,104,141,142^ In these studies, *Lactobacillus* administration in animal models of MDD characteristics show behavioral changes including reduced anxiety, depression,^4,9,104^ reduced inflammation,^170^ and improved metabolism.^104^ In one study, GABA producing *L. brevis* reduced depressive-like behavior and corticosterone production, which correlated with increased intestinal GABA concentration, in a mouse model of metabolic syndrome.^104^ In another study, this time in healthy mice, treatment with GABA producing *L. rhamnosus* induced region-specific changes in GABA_A_ and GABA_B_ receptors in the hippocampus, amygdala, and *locus coeruleus* in the brain, reduced corticosterone production, and reduced depressive-like behavior.^9^ Subdiaphragmatic vagotomy reversed the protective effects of *L. rhamnosus* on depression and anxiety-like behaviors and expression of GABA_A_ and GABA_B_ receptors, highlighting the role of the vagus nerve in the gut-microbiome-brain axis.^9^ Studies using exogenous GABA producing *Lactobacillus* administration are beginning to emerge in AD literature. One such study suggested that administration of *Lactobacillus* prior to development of pathologies, when other studies in mice have shown *Lactobacillus* to be depleted in mice modeling AD pathologies,^22,28^ can delay or reduce pathology burden.^126^ Species in the genus *Lactobacillus* are emerging as key players in the gut-microbiome-brain axis. In the future, *Lactobacilli* may be leveraged to target the gut microbiome via GABAergic signaling pathways early in AD.

## Conclusion

In this review, we explored potential effects of microbe-derived GABA epithelial barrier integrity as a potential mechanism underlying the gut-microbiome-brain axis in AD. We highlight the contribution of gut microbial communities for their role in gut barrier integrity and neurotransmitter synthesis. Emerging evidence suggests that AD, a complex neurodegenerative disease, is associated with preclinical changes in the gut microbiome, potentially allowing damage to gut epithelial cells and an altered neurotransmitter profile. Increased GABAergic microbes in the gut after AD onset may be the body’s attempt to increase ambient GABA. This however, may be a double-edged sword, as increased GABA may also increase gut permeability, allowing translocation of harmful microbial products across the gut and BBB. This temporal interaction between tight junctions in the gut and brain may be, in part, mediated by microbial metabolites such as GABA. To date, little research has been conducted on the effects of GABA on the mucin layer and tight junction integrity in AD. Future research should seek to understand the disease-specific mechanisms by which microbial derived GABA interacts with gut barrier integrity and AD.
